# RAD52: Paradigm of Synthetic Lethality and New Developments

**DOI:** 10.3389/fgene.2021.780293

**Published:** 2021-11-23

**Authors:** Matthew J. Rossi, Sarah F. DiDomenico, Mikir Patel, Alexander V. Mazin

**Affiliations:** Department of Biochemistry and Structural Biology, University of Texas Health Science Center, San Antonio, TX, United States

**Keywords:** Rad52, homologous recombination, single strand annealing, break induced replication, synthetic lethality

## Abstract

DNA double-strand breaks and inter-strand cross-links are the most harmful types of DNA damage that cause genomic instability that lead to cancer development. The highest fidelity pathway for repairing damaged double-stranded DNA is termed Homologous recombination (HR). Rad52 is one of the key HR proteins in eukaryotes. Although it is critical for most DNA repair and recombination events in yeast, knockouts of mammalian RAD52 lack any discernable phenotypes. As a consequence, mammalian RAD52 has been long overlooked. That is changing now, as recent work has shown RAD52 to be critical for backup DNA repair pathways in HR-deficient cancer cells. Novel findings have shed light on RAD52’s biochemical activities. RAD52 promotes DNA pairing (D-loop formation), single-strand DNA and DNA:RNA annealing, and inverse strand exchange. These activities contribute to its multiple roles in DNA damage repair including HR, single-strand annealing, break-induced replication, and RNA-mediated repair of DNA. The contributions of RAD52 that are essential to the viability of HR-deficient cancer cells are currently under investigation. These new findings make RAD52 an attractive target for the development of anti-cancer therapies against BRCA-deficient cancers.

## Introduction

Rad52 was first identified along with a large group of homologous recombination (HR) proteins in a screen for DNA-repair deficient *S. cerevisiae* mutants following ionizing radiation (Game and Mortimer 1974). These proteins (which include Rad52, Rad50, Rad51, Rad54, Rad55, Rad57, Rad59, Rdh54, Mre11, and Xrs2) were collectively called the RAD52 epistasis group genes because of all these genes, *Δrad52* displayed the most severe defect in double-strand break (DSB) repair. Furthermore, RAD52 appeared to be critically important for most, if not all, recombination events in yeast including meiotic recombination, homologous DNA integration, and mating-type switching (Malone et al., 1980; Symington 2002). In contrast, the role of mammalian RAD52 has been largely unexplored due to the lack of a DNA repair or recombination phenotype in RAD52-deficient cells. However, recent discoveries point to multiple novel and intriguing functions of RAD52 in mammalian cells. Recent works have shown that because of RAD52’s important role in various aspects of the DNA damage response (DDR), RAD52 mutations can cause synthetic lethality in cells deficient in BRCA1, BRCA2, PALB2, or RAD51C genes. Deficiencies in these genes are responsible for nearly half of hereditary breast and ovarian cancers, and ovarian cancers and for a significant fraction of prostate and pancreatic cancers (Feng et al., 2011; Nogueira et al., 2019; Gottifredi and Wiesmuller 2020). Therefore, Rad52 has potential as a therapeutic target in the treatment of these and some other cancers. Here, we will focus on the recent advancements in understanding RAD52’s role in various DNA repair pathways, and on the work that is underway to develop RAD52 inhibitors that can serve as cancer therapeutics.

### Overview of RAD52 Functions in DNA Repair

**FIGURE 1:**
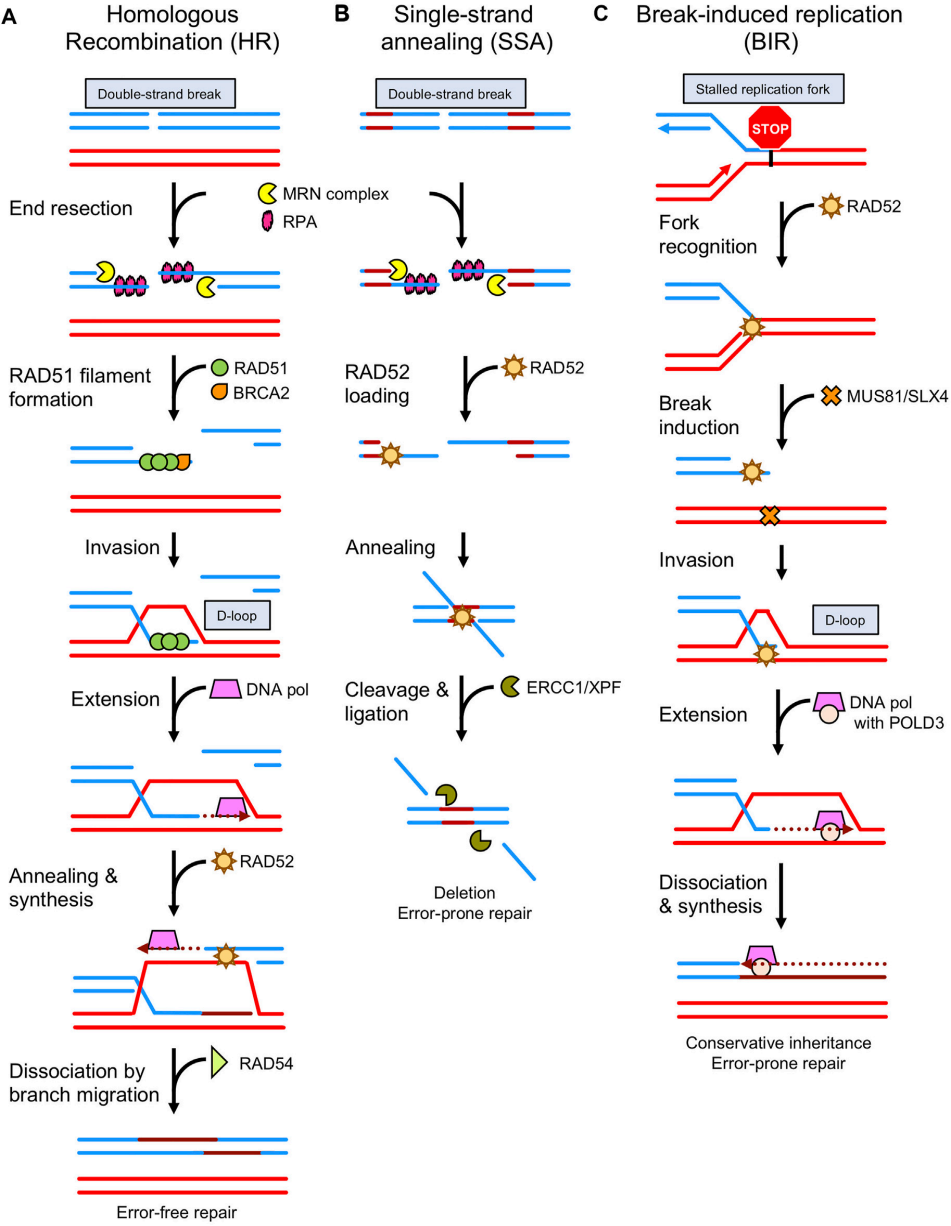
Comparison of RAD51 and RAD52-mediated DNA repair pathways. **(A)** During homologous recombination (HR), the ends of the double strand break (DSB) are resected by nucleases (ex. MRN complex) [see (Zhao et al., 2020) for mechanism of action], exposing single-strand DNA (ssDNA) that becomes bound by RPA. Then the mediator protein, BRCA2 initiates loading of RAD51 on ssDNA helping to displace RPA. RAD51 oligomerizes, forming a nucleoprotein filament, and then searches for the homologous DNA sequence on the intact chromosome. The RAD51 filament invades the intact dsDNA to form a D-loop structure. Further processing by DNA polymerases, chromatin remodelers (ex. RAD54), nucleases, and ligases restore the intact DNA sequence through error-free repair. **(B)** Alternative to HR, single strand annealing (SSA) begins after resection with the binding of RAD52 to ssDNA. RAD52 promotes the annealing of exposed homologous ssDNA regions on either side of the DSB. Processing of the annealed DNA by nucleases (ex. ERCC1/XPF) results in error-prone repair as the sequences between homologous regions are lost. **(C)** RAD52 also recognizes and repairs stalled replication forks *via* break-induced replication (BIR). The structure is cleaved by the endonuclease complex MUS81 and processed by EEPD1 (Kim et al., 2017; Sharma et al., 2020). Bound to the one-ended DNA break, RAD52 invades the dsDNA to form a D-loop. The DNA polymerase contains a non-enzymatic subunit, POLD3, that appears to be specific to this type of repair.

Genomic DNA is under constant attack by endogenous metabolic byproducts, exogenous chemicals, and environmental stress such as ultraviolet radiation. In response, cells have developed numerous DNA protective and repair mechanisms to maintain genome stability. Mis-repaired DNA damage can be mutagenic and lead to cancer (Hoeijmakers 2009). One of the most harmful types of DNA damage is the DSB, and the most accurate way to repair DSBs is through the HR pathway. The salient step of HR is performed by the recombinase protein RAD51 in conjugation with auxiliary proteins. Following DNA replication and the formation of a sister chromatid, RAD51 will bind the resected, single-stranded DNA (ssDNA) end of a DSB, form a nucleoprotein filament, and search for the homologous DNA sequence on the intact sister chromatid (Figure 1A). To gain access to the resected end, RAD51 must compete with the ssDNA-binding protein replication protein A (RPA) that is ubiquitous in all eukaryotes. To successfully compete with RPA’s high affinity for ssDNA, RAD51 requires a mediator protein (Sung 1997; Kowalczykowski 2015). The major mediator in budding yeast is Rad52, which promotes the displacement of RPA by Rad51 (Sung and Klein 2006). In mammals, that major RAD51 mediator role is filled by BRCA2 (Esashi et al., 2007; Zelensky et al., 2014; Scully et al., 2019). Nonetheless, mammalian RAD52 retains an ability to physically interact with RAD51 and RPA, but the role of these interactions is a matter of investigation.

The RAD51 filament searches for a homologous DNA on the sister chromatid and performs strand exchange to produce a joint molecule also known as a D-loop. From the D-loop, DNA polymerase then uses the homologous DNA strand as a template and the 3′-end of the broken DNA strand as a primer to commence DNA repair synthesis. The second end of the DSB is captured by RAD52 and annealed to the displaced strand of the D-loop to provide the template for the second strand synthesis. Upon completion of DNA synthesis, the D-loops can be dissociated by RAD54, an ATP-dependent motor protein that interacts with RAD51 and promotes branch migration, or by helicases like BLM (van Brabant et al., 2000; Bugreev et al., 2006; Bugreev et al., 2007). DNA is extended by DNA polymerase and then annealed to the ssDNA portion of the second broken DNA end; followed by gap filling, flap removal, and nick sealing by DNA polymerases. Flap nucleases and ligases then restore the original DNA sequence (Kawale and Sung 2020).

From an accuracy standpoint, it is preferable to repair all DNA damage through HR. However, a preference for sister chromatid limits most HR activity to the late S/G_2_ phase of the cell cycle. Additionally, the HR mechanism is time consuming. Even when operating at full capacity, RAD51-dependent HR can only handle ∼5 DSBs in a cell at once (Mladenov et al., 2020). In order to repair the ∼50 DSBs a normal cell suffers through one cell cycle (Hoeijmakers 2009), the cell relies on another pathway termed classical non-homologous DNA end joining (c-NHEJ) (Vilenchik and Knudson 2003). Here, the Ku70/80 heterodimer, DNA-PKcs and DNA Ligase IV with several auxiliary proteins promote re-joining of DNA ends by ligation (Chang et al., 2017; Ghosh and Raghavan 2021). c-NHEJ is rapid and efficient; requires no homologous sequences and repairs the break with minimal loss of DNA sequence. Currently, there is no known role of RAD52 in c-NHEJ.

At times of high DSB stress (such as during DNA replication), repair of DSBs may also be processed through the alternative DNA end joining (a-EJ) and single-strand annealing (SSA) pathways. In a-EJ, the MRN-CtIP nuclease complex generates short (<20 bps) resected ends at the DSB (Bhargava et al., 2016; Chang et al., 2017). Poly-ADP ribose polymerase-1 (PARP1) and DNA polymerase θ anneal microhomologies (∼10 bp) between DNA ends, followed by XRCC1- and DNA ligase III-mediated end processing (Srinivasan et al., 2019) that generate an intact DNA molecule. a-EJ was reported to be partially dependent on RAD52, likely through RAD52’s annealing activity (Kan et al., 2017; Hendrickson 2020), which prevents premature usage of a-EJ until the cell enters mitosis (Llorens-Agost et al., 2021). RAD52 also inhibits PARP-mediated single-strand break repair by interfering with colocalization of XRCC1 and DNA ligase III (Wang et al., 2021).

RAD52 plays a major role during SSA. Similar to the main HR mechanism, DSB ends in SSA are resected by helicases (BLM and WRN) and nucleases (DNA2, CtIP, and EXO1) to generate long segments of ssDNA (Ceccaldi et al., 2016). Then RAD52 protein binds to the resected DNA ends (Hanamshet et al., 2016) and promotes the annealing of ssDNA regions of homology (>30 bps) (Figure 1B). Following annealing, the ERCC1-XPF complex binds the N-terminal domain of RAD52 to attenuate the SSA activity of RAD52, while enhancing its own endonuclease activity (Motycka et al., 2004). The RAD52-ERCC1-XPF complex localizes to the repair-intermediate and cleaves the 3’ ssDNA tails that resulted from RAD52 annealing the homologous sequences together. Gaps are filled by unidentified polymerases and the DNA ends are joined by DNA ligase I (Bhargava et al., 2016). During processing in a-EJ and SSA, one of the two original homologous regions, along with the intervening DNA, are deleted. Thus, in contrast to HR, these alternative pathways are error-prone/mutagenic. It was shown that SSA could cause interchromosomal translocations between two DSBs occurring simultaneous between two different sets of repeat elements. In this case, SSA resulted in the loss of one repeat on each chromosome (Elliott et al., 2005).

New focus was directed toward the a-EJ and SSA repair pathways once it was shown that PARP inhibitors were effective in treating BRCA-deficient cancers. BRCA-deficient cancer cells are defective in HR. As a consequence, they become dependent on other DNA repair pathways for their survival. PARP1 inhibitors are a clinically approved treatment for certain types of BRCA-deficient cancers (Myers et al., 2020). Recent works have shown that because of its important role in various aspects of the DDR, RAD52 also has potential as a therapeutic target in the treatment of hereditary breast, ovarian, and some other cancers (Feng et al., 2011; Nogueira et al., 2019; Gottifredi and Wiesmuller 2020).

### The Biochemical Activities of RAD52

Human RAD52 is a 418 amino acid (46 kDa) protein with two domains. The N-terminal domain contains two DNA binding domains and is highly conserved among eukaryotes (42% identity between *H. sapiens* and S. *cerevisiae* homologs) (Hanamshet et al., 2016). The crystal structure of this highly stable domain showed that the RAD52 N-terminal domain oligomerizes to form an undecameric ring structure (Kagawa et al., 2002; Singleton et al., 2002). The base of this ring forms a large, positively charged channel that accommodates ∼40 nt of ssDNA per ring. RAD52 promotes ssDNA annealing (Mortensen et al., 1996; Kagawa et al., 2001; Khade and Sugiyama 2016; Saotome et al., 2018). RAD52-mediated ssDNA annealing persists in the presence of RPA (Sugiyama et al., 1998), and is essential to RAD52’s ability to perform SSA repair. A secondary DNA binding site runs parallel to the primary ssDNA binding site at the outer portion of the ring structure. This site accommodates double-stranded DNA (dsDNA) or ssDNA, plays a role during ssDNA annealing, and allows RAD52 to perform DNA strand exchange (Kagawa et al., 2008). Like RAD51, RAD52 can promote the formation of a D-loop between ssDNA and plasmid DNA (Kagawa et al., 2001). Through its two DNA binding sites, RAD52 binds the one-ended DSB and performs strand exchange to produce a D-loop structure in a mechanism termed break-induced replication (BIR) (Figure 1C) (Kagawa et al., 2001; Llorente et al., 2008). This activity is abrogated when either DNA binding site is inactivated through mutation (Hanamshet and Mazin 2020). The break is then repaired by POLD3-dependent DNA synthesis (Lemacon et al., 2017). Unlike RAD51, RAD52 does not form long filamentous structures on ssDNA and does not hydrolyze ATP; instead RAD52 forms large co-aggregated stacked ring structures through its C-terminal domain (Ranatunga et al., 2001) that facilitate ssDNA annealing (Kagawa et al., 2008; Saotome et al., 2018).

The C-terminal domain of RAD52 also contains regions that bind to RPA (Shinohara et al., 1998) and RAD51 (Shen et al., 1996). Although human RAD52 binds directly to RPA, this interaction is not essential for the major functions of RAD52 in DNA repair, as the RAD52 N-terminal domain alone was sufficient to maintain viability of BRCA-deficient cells (Hanamshet and Mazin 2020). In yeast Rad52, the binding to RPA is involved in the mediator function of Rad52. Yeast Rad52 binds both RPA and Rad51, which results in the displacement of RPA from resected ssDNA ends and the promotion of Rad51 nucleoprotein filament formation (Sung 1997; New et al., 1998; Shinohara et al., 1998; Gibb et al., 2014; Ma et al., 2017). The role of human RAD52 interactions with RAD51 and RPA remains to be fully understood. We showed that RPA-RAD52 interaction is required for stimulation of RAD52’s inverse RNA strand exchange activity by RPA (Mazina et al., 2017).

### RAD52 During DDR Pathway Choice

Understanding the rules governing the competition and cooperation between c-NHEJ, HR, SSA, and a-EJ to repair DSBs remains an open research topic. Extensively resected DNA ends act as a signal to promote RAD51-directed repair and suppress c-NHEJ. By default, p53 binding protein 1 (53BP1) suppresses the end resection activity of the MRE11-RAD50-NBS1 complex (MRN) to limit HR during G_1_ phase. But once the cell enters S phase, ataxia-telangiectasia mutated (ATM) kinase is recruited to the damage site through an interaction with MRN and activated through autophosphorylation at Ser 1981 (Shiloh and Ziv 2013). ATM then phosphorylates other target proteins such as histone H2AX on Ser139 (γ-H2AX). This phosphorylation event stimulates the recruitment of BRCA1 (Delia and Mizutani 2017). BRCA1 interacts with MRN and CtIP to promote extensive end resection by the exonucleolytic complex EXO1-DNA2 and expose 3’ ssDNA ends (Reginato and Cejka 2020).

Following resection, mediator proteins including RAD52, PARP1, and BRCA2 compete with each other and the DNA-damage sensing proteins previously recruited to the site of damage. This competition is partially modulated through cellular signals transduced through posttranslational modifications. The histone acetyltransferase p300/CBP plays a role in regulation of DNA transcription, replication, and repair (Dutto et al., 2018). For instance, it acetylates histones to relax chromatin and increase DNA accessibility to other proteins. RAD52 is also acetylated by p300/CBP at DSB sites, and deacetylated by SIRT2/SIRT3 (Yasuda et al., 2018). The acetylated form of RAD52 persisted at sites of DNA damage longer compared to an acetylation-deficient RAD52 mutant containing ten arginine substitutions. This RAD52 mutant also decreased the ability of RAD51 foci to be retained at DSB sites. A RAD52 acetylation-mimic mutant containing ten glutamines had a higher affinity for RAD51 and RPA in a yeast two-hybrid system. It was speculated that competition between RAD52 and BRCA2 allowed RAD51 nucleoprotein filament expansion following initiation by BRCA2 (Yasuda et al., 2018). In this scenario, RAD52 acetylation would act as a signal to promote homology-directed repair pathways.

DSS1 (Sem1 in yeast) is a small, highly acidic protein that binds BRCA2 and stimulates RAD51 filament formation (Liu et al., 2010). More recently, it was also discovered to bind RAD52 and stimulate its ssDNA annealing and D-loop formation activities (Stefanovie et al., 2020). DSS1 does not appear to bind DNA on its own; instead it enhances the ssDNA binding activities of BRCA2 and RAD52 to facilitate the initial steps of DSB repair (Zhao et al., 2015).

RAD52 activities are also modulated by several phosphorylation events. Cyclin-dependent kinase 1 (CDK1) regulates the transition through the cell cycle by associating with phase-specific cyclins. In yeast, the homolog of CDK1, Cdc28, coupled with Clb2 or Clb3 cyclins phosphorylates Rad51 at S125 and S375 to increase binding affinity for ssDNA; and Rad52 at Thr412 to promote RAD52 oligomerization (Lim et al., 2020). These residues are conserved from yeast to human (Hanamshet et al., 2016; Kelso et al., 2017), but it remains to be seen if these modifications occur in higher eukaryotes. In humans, RAD52 is phosphorylated at Y104 by the ATM-activated c-ABL kinase. This modification enhances RAD52’s ssDNA annealing activity by inhibiting DNA binding at the secondary site (Honda et al., 2011).

The mechanism through which signaling specifies a repair pathway is not understood, but one determining factor appears to be the level of DNA damage. During G_2_ phase, RAD51-dependent HR can only function efficiently at the low DSB load that is typical under normal cell growth. RAD51 binding to chromatin saturates at ∼20% total RAD51 even at high levels of ionizing radiation (Mladenov et al., 2020). It was shown *in vivo* that efficient RAD51 foci formation at DSBs depended on the prerequisite binding of 53BP1 (Ochs et al., 2016). Exhaustion of 53BP1 (as occurs at high DSBs) limited the RAD51’s ability to form stable foci. As a consequence, HR does not significantly contribute to repair when the cell is overloaded with DSBs. In cells experiencing high DSB (∼50 simultaneous DSBs), HR handles about 5 repairs at once (10%). While RAD51-dependent repair is suppressed at high DSB, end resection is not. To back up the saturated RAD51 HR pathway, the RAD52-dependent SSA pathway becomes activated. This activation can be achieved through competition between 53BP1 and the E3 ubiquitin ligase protein RNF169. Overexpression of RNF169 or knockout of 53BP1 or BRCA2 in reporter cell lines results in hyperactive SSA repair (Tutt et al., 2001; An et al., 2018). The SSA pathway is activated at IR doses up to 4-fold higher than the saturation level for HR. Above that point RAD52 is also suppressed, leaving only c-NHEJ to repair DSBs (Mladenov et al., 2020).

### RAD52 in Protection and Processing of Stalled Replication Forks

**FIGURE 2:**
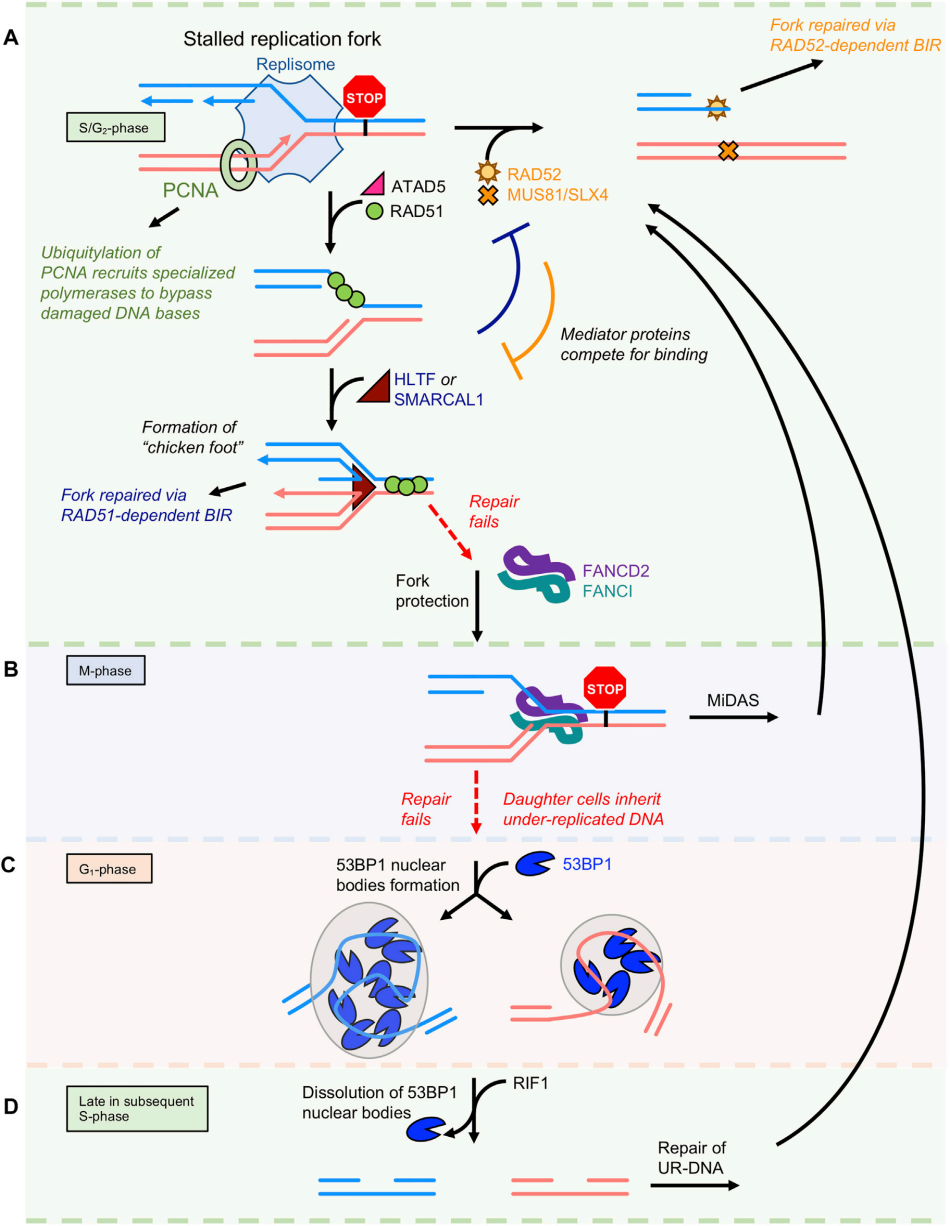
Repair of stalled replication forks *via* BIR. **(A)** Multiple DNA repair pathways compete to repair stalled replication forks during S/G_2_ phase of the cell cycle. **(B)** Once the cell enters M-phase, unrepaired forks become bound by the FANCD2/FANCI complex. It will attempt to repair the lesion again by a RAD52-dependent BIR-like pathway termed mitotic DNA synthesis (MiDAS). **(C)** If still unsuccessful, the cell with complete mitosis with each daughter cell inheriting under-replicated ssDNA that is protected by the 53BP1 protein during G_1_. **(D)** In the subsequent S-phase, the cell has one final attempt to repair under-replicated DNA *via* BIR. After this point, the cells must undergo apoptosis or pass on an incomplete genome.

During DNA replication, the replisome encounters many roadblocks. The cell has developed several complimentary and competing pathways to recover from the DNA lesions that stall replication forks (Kondratick et al., 2021). Restart of stalled replication forks is complex and fraught with pitfalls that contribute to genomic instability and disease progression in humans (Neelsen and Lopes 2015). A wide range of proteins are recruited to stalled replication forks including ssDNA binding proteins and recombinases (RPA, BRCA2, RAD51, RAD52, RADX), translocases (SMARCAL1, ZRANB3, HLTF, SHPRH, WRN, RECQ1, ATAD5), and endo/exonucleases (MRE11, EXO1, DNA2, MUS81) (Kondratick et al., 2021; Nickoloff et al., 2021). An active area of research is aimed at understanding the interplay between these factors. Depending on the type of block, stalled replication forks can be repaired through several mechanisms (Figure 2A). Damaged DNA bases are bypassed *via* translesion synthesis, in which specialized DNA polymerases are recruited to the fork by the ubiquitylation of PCNA. These polymerases have low fidelity for base pairing, allowing them to bypass DNA lesions at the expense of potential mutagenesis. In yeast, Rad52 recruits the E2/E3 ligases Rad6/Rad18 to ubiquitylate PCNA (Cano-Linares et al., 2021).

DNA replication stress often leads to the uncoupling of leading and lagging strand synthesis and the accumulation of ssDNA gaps (Zellweger et al., 2015). These types of stalled replication forks are repaired by BIR, in which the ATAD5-RLC removes PCNA and recruits RAD51 (Park et al., 2019). RAD51 filament protects the fork through a mechanism that does not require its ATPase activity (Mason et al., 2019), and presumably recruits translocases, such as RAD54 (Bugreev et al., 2011), SMARCAL1, and/or ZRANB3 (Kondratick et al., 2021) that reverse replication forks and create a “chicken-foot” structure that is cleaved by MUS81 to generate a one-ended DSB.

First described in recombinant dependent replication of bacteriophage T4 (Luder and Mosig 1982), and later in yeast (Morrow et al., 1997), BIR’s role in mammalian systems is only now beginning to be appreciated (Costantino et al., 2014). The molecular mechanism of BIR has been extensively studied in yeast systems (Malkova and Ira 2013). At the one-ended DSB, the end is resected and Rad52 initiates formation of a Rad51 nucleoprotein filament on ssDNA. It invades the homologous region of the intact sister strand to form a D-loop. Then a replisome assembles containing a non-essential subunit of DNA polymerase δ called Pol32 (Lydeard et al., 2007). Unique to BIR, the D-loop then moves with the replication fork during leading strand synthesis (Smith et al., 2007). Lagging strand synthesis is delayed until the sister chromatin separates, resulting in conservative DNA replication (as opposed to traditional semi-conservative) (Wilson et al., 2013). The recently discovered RADX protein binds ssDNA and directly interacts with the RAD51 to destabilize the nucleofilament and ensure that the resumed DNA replication proceeds at the proper rate (Adolph et al., 2021).

When the DNA damage load overwhelms RAD51’s capabilities, then collapsed replication forks are restarted by the RAD52-dependent BIR pathway. This pathway has been studied in BRCA2-deficient cells where the RAD51 pathway is no longer viable. In this environment, fork reversal is deregulated and leads to excessive degradation by MRE11 (Mijic et al., 2017; Taglialatela et al., 2017). The exonuclease activities of MRE11 and EXO1 generate extensive ssDNA that increases chromosome breaks and genome instability. These partially resected forks are cleaved by MUS81 to create one-ended DSBs. In CHK1-deficient cells where the G_2_/M cell cycle checkpoint is lost, cell survival is dependent on RAD52 and MUS81 to relieve replication stress by creating DSBs as the cell tries to complete the cell cycle (Murfuni et al., 2013).

In Rad52-dependent BIR (Malkova et al., 1996), ssDNA annealing by Rad52 and Rad59 are responsible for pairing homologous sequences. It is also possible the DNA pairing (D-loop formation) activity of Rad52 plays a role in BIR initiation. In yeast, Rad59 removes the inhibitory effect of Rad51 on Rad52’s ability to anneal ssDNA and promote single-strand template repair (Gallagher et al., 2020). Rad52-dependent BIR also requires the translocase protein Rdh54 and the exonuclease/resolvase complex MRX (Signon et al., 2001) to complete the process. Rad52-mediated BIR in yeast is highly mutagenic due to high level of template switching during replicative repair (Kockler et al., 2021). Recent studies suggest that RAD52-driven BIR may promote genome instability in human cancers. The Halazonetis group used the overexpression of oncogenic cyclin E in U2OS cells to induce DNA replication stress and identify POLD3 or POLD4 (homologs of yeast Pol32), MUS81 and SLX4 (endonuclease complex), and RAD52 as required for BIR (Costantino et al., 2014; Sotiriou et al., 2016).

There exist difficult-to-replicate regions of the genome termed common fragile sites. They tend to be at AT-rich sequences in long coding regions where transcribing RNA polymerases inevitably collide with replicating DNA polymerases (Helmrich et al., 2011). An under-replicated DNA (one copy instead of two) event probabilistically occurs at least once a cell cycle (Al Mamun et al., 2016). At colliding polymerases, the forks stall and become bound by the FANCD2/FANCI complex that tether sister chromatids to each other (Figure 2B). The cell attempts to repair these DNA lesions *via* mitotic DNA synthesis (MiDAS). The mechanism of MiDAS appears equivalent to BIR, as it produces conservative DNA replication and requires MUS81-EME1, SLX4, POLD3, and RAD52 (Al Mamun et al., 2016).

When MiDAS fails to repair the damage before cell division, then the daughter cells inherit under-replicated DNA marked as lesions sequestered during G_1_ phase by 53BP1 nuclear bodies (Lukas et al., 2011) (Figure 2C). Late in the subsequent S-phase, 53BP1 nuclear bodies dissolve by the RIF1-mediated activation of late replication origins. This triggers recruitment of RAD52 and gives the cell a second chance to repair the damage (Spies et al., 2019) through a BIR-equivalent pathway (Figure 2D). Many questions remain regarding the signaling and molecular mechanisms that govern the repair of known fragile sites (Bertolin et al., 2020). If these events are as common as the literature suggests, how can their repair rely on error-prone RAD52-dependent BIR mechanisms? How would these genes survive multiple generations if they are prone to break, and repair results in DNA sequence loss?

### RAD52 in RNA-Dependent DNA Repair

**FIGURE 3:**
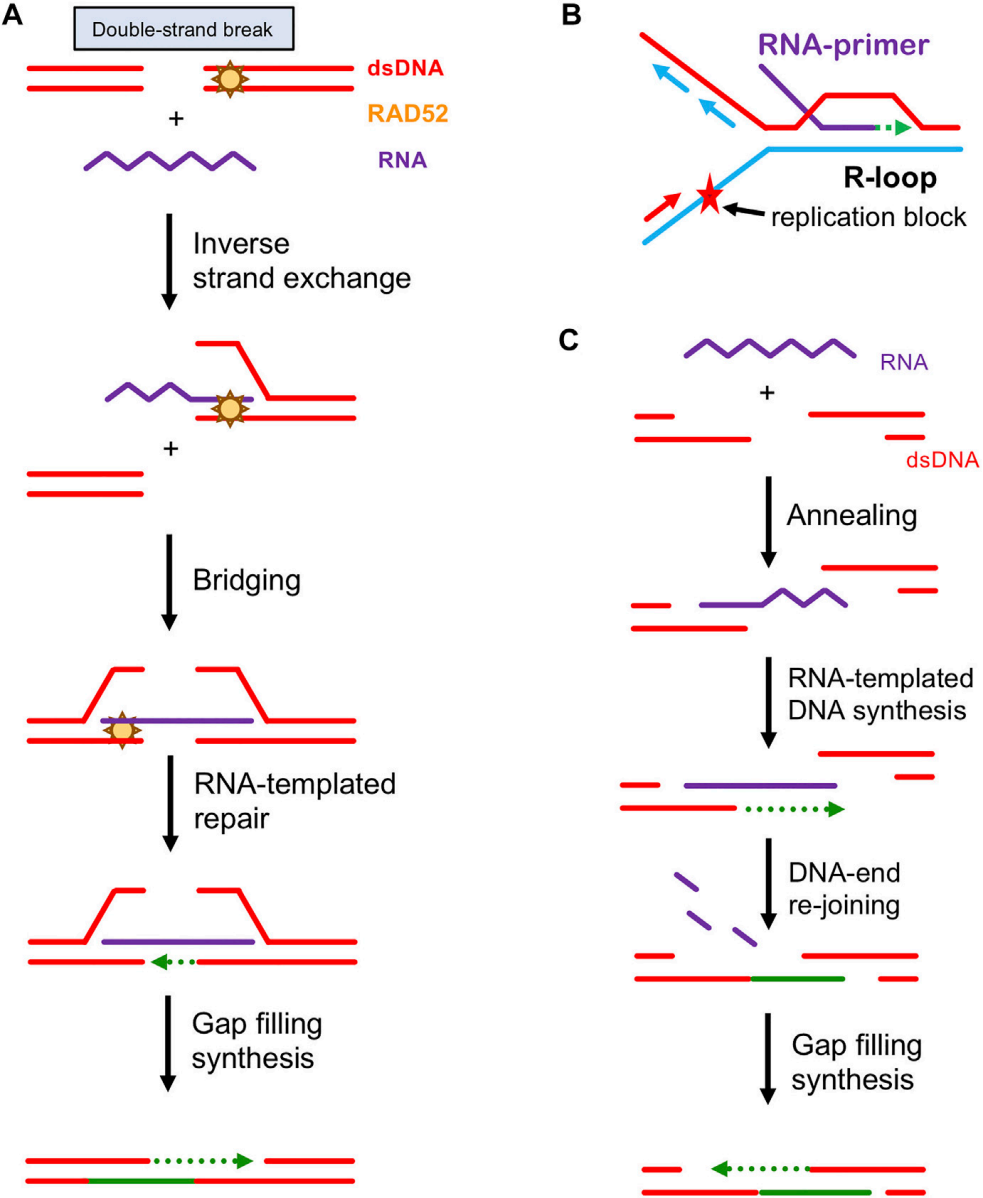
Proposed Mechanisms of RNA-Dependent DSB Repair. **(A)** Repair of DSBs *via* inverse RNA strand exchange. Rad52 forms a complex with DSB ends either blunt ended or minimally processed by exonucleases/helicases. Then, RAD52 promotes inverse RNA strand exchange with a homologous RNA transcript. The RNA transcript in the resultant DNA:RNA hybrid provides a template for DNA repair synthesis. The single-stranded tails are removed by flap nucleases, the gaps are filled in, and any remaining nicks are sealed by DNA ligases, restoring the original DNA sequence in an error-free manner. **(B)** Restart of DNA synthesis stalled at DNA damaged site primed by an R-loop. **(C)** A tentative role of RAD52 annealing activity in DSB repair. RAD52 promotes annealing between the ssDNA ends of an exonucleolytically processed DSB and homologous RNA transcript. The RNA transcript provides a template for DNA repair synthesis that extends the ssDNA end ensuring an overlap with the ssDNA of another DSB end. This is followed by re-joining of the DSB ends *via* ssDNA annealing, removal of DNA:RNA heteroduplex by RNase H, filling the gaps by DNA polymerases and sealing the nicks by DNA ligases.

HR is known to use homologous DNA sequences as a template to carry out high-fidelity repair of DSB and other lethal lesions. However, recent data shows that HR can also use a homologous RNA transcript to repair DSB damage (Keskin et al., 2014; Mazina et al., 2017; Michelini et al., 2018). This defies the central dogma, in which genetic information flows from DNA to RNA. Strong support for the use of an RNA template in HR came from experiments in *Saccharomyces cerevisiae*. Keskin et al. developed a DSB-inducible system to monitor repair by a homologous RNA transcript (Keskin et al., 2014). They showed that RNA can be directly used as a template for DSB repair in the absence of reverse transcriptases. Further, the efficiency of this process increased dramatically in the absence of RNase H. It was proposed that upon DSB formation at an actively transcribed locus, the homologous RNA transcript forms a DNA:RNA heteroduplex intermediate that bridges the two DNA ends together and serves as a template for gap filling synthesis (Keskin et al., 2014; Mazina et al., 2017; Michelini et al., 2018) (Figure 3A).

In addition to this bridging-template mechanism, RNA transcripts were also implicated in DNA replication-restart*.* RNA is known to form R-loops with homologous DNA, the three-stranded structures consisting of an RNA-DNA hybrid and the displaced ssDNA strand. Thus, up to 5% of human and 8% of yeast genome is susceptible to DNA:RNA hybrid or R-loop formation (Chedin 2016; Wahba et al., 2016). It was proposed by Kogoma that R-loops may prime a restart of DNA replication forks stalled at damaged DNA in *E. coli* (Kogoma 1997) (Figure 3B). While DNA repair by canonical HR requires sister chromatids as a source of homologous DNA template sequences and therefore is limited to S/G_2_ phase, RNA-dependent DNA repair may occur in non-dividing cells, like terminally differentiated neurons (Welty et al., 2018).

Rad52 was implicated in RNA-dependent DSB repair by genetic data from *S. cerevisiae* (Keskin et al., 2014; Mazina et al., 2017). Rad52 knockouts in yeast reduced the level of RNA-dependent DNA repair. The role of RAD52 in RNA-dependent DSB repair is also supported by data from human cells (Wei et al., 2015; Yasuhara et al., 2018). Currently, the function of RAD52 in RNA-dependent DSB repair is under intense investigation. We recently reported an unconventional type of strand exchange, known as inverse strand exchange, that yeast and human Rad52 promote between RNA and homologous dsDNA (Mazina et al., 2017) (Figure 3A). This activity is different from the conventional (forward) strand exchange activity of major recombinases of the RAD51 family. In case of RAD51, the active species in DNA strand exchange is a nucleoprotein filament that RAD51 forms with ssDNA. The filament binds dsDNA to promote the search for homology and strand exchange. In contrast, RAD52 forms the active nucleoprotein complex with dsDNA which promotes strand exchange with free RNA or ssDNA. The bacterial DNA repair protein, RecA, was first discovered to have this type of DNA strand exchange (Zaitsev and Kowalczykowski 2000). In eukaryotes, this activity is unique to Rad52, neither the major recombinase Rad51 nor the yeast Rad52 paralog Rad59 perform inverse RNA strand exchange. These biochemical results are consistent with genetic data in *S. cerevisiae*, which show that RNA-templated DSB repair is dependent on Rad52 but not on Rad1, Rad9, or on end resection factors Sae2, Exo1, and Mre11 (Mazina et al., 2017; Meers et al., 2020). Moreover, the RAD52 R55A mutant defective in inverse RNA strand exchange fails to promote RNA-dependent DNA repair in budding yeast. Recently, it was found that RNA-templated DNA repair occurs in yeast cells through two mechanisms: DSB-dependent and DSB-independent (Meers et al., 2020). Only the DSB-dependent mechanism requires RAD52, which is consistent with the RNA inverse strand exchange activity of RAD52 that occurs in the proximity of DNA ends. Overall, genetic data in *S. cerevisiae* support the biological role of inverse RNA strand exchange *in vivo*.

In addition to inverse RNA and DNA strand exchange, Rad52 is known to promote annealing between complementary ssDNA molecules (Mortensen et al., 1996). More recently it was found that RAD52 can also promote annealing between ssDNA and complementary RNA (Keskin et al., 2014; McDevitt et al., 2018). It was suggested that this RNA/DNA annealing activity may also contribute to DSB repair by bridging the exonucleolytically processed DNA ends (Figure 3C).

RNA transcripts can be transcribed by reverse transcriptases encoded by retrotransposons or retroviruses. Genetic data in *S. cerevisiae* show that the resultant cDNA may be used efficiently for DSB repair *via* the conventional RAD51-dependent HR mechanisms (Keskin et al., 2014). In the absence of reverse transcriptases, short DNA synthesis on RNA templates can be carried out by DNA polymerases, which have limited reverse transcriptase activity. It was shown that several polymerases including yeast replicative polymerases (δ and α) possess minimal reverse transcriptase activity *in vitro* (Storici et al., 2007). Human Pol η and Pol θ are capable of utilizing an RNA template (Su et al., 2019; Chandramouly et al., 2021). Recently, it was shown that yeast Pol ζ is required for RNA-dependent DNA repair (Meers et al., 2020). In yeast, it was proposed that as DNA Pol δ encounters a DSB at an actively transcribed locus, Rad52 generates an DNA:RNA heteroduplex (R-loop) at the proximity of the DSB. Then, polymerase switching occurs and the RNA in this heteroduplex is used as a template for repair by Pol ζ (Meers et al., 2020).

Several recent reports linked the function of RAD52 in human cells to a specific type of HR occurring within transcriptionally active genome regions. This type of HR was named transcription-coupled homologous recombination (TC-HR) (Welty et al., 2018) or transcription-associated homologous recombination repair (TA-HRR) (Yasuhara et al., 2018). It was found that several HR proteins including RAD52, RAD51, RAD51C and RPA form a larger number of nuclear foci in response to DNA damage in active transcription regions (Wei et al., 2015). In contrast, several other HR proteins like NBS1, BRCA1, and BRCA2; or NHEJ proteins Ku70 and DNA ligase IV did not show such preference for foci formation in active transcription regions. Unlike canonical HR that occurs in S/G_2_ cell cycle phase, TC-HR can also operate in G_0_/G_1_ phase (Welty et al., 2018).

It was found that RAD52 recruitment to DNA damage sites occurs in a DNA:RNA hybrid-dependent manner during TC-HR (Wei et al., 2015; Yasuhara et al., 2018). Inhibition of transcription at the site of DNA damage or overexpression of RNase H reduced RAD52 recruitment. Also, it was suggested that RAD52 can be recruited through direct binding to DNA:RNA hybrids or R-loops (Yasuhara et al., 2018). While RAD52 can indeed bind to these structures, its preferential substrate is ssDNA, not DNA:RNA hybrids (Mazina et al., 2017; Welty et al., 2018). On the other hand, the preferential binding of RAD52 to the ssDNA strand displaced in R-loops, does not seem strong enough to support that as a mechanism of RAD52 recruitment. Recently, it was shown that RAD52 displays an increased affinity for DNA: RNA hybrids containing m5C-modified RNA *in vitro*; m5C(s) are generated in mRNA by the RNA methyltransferase TRDMT1 that is recruited to DNA damage sites (Chen et al., 2020). Additional quantitative characterization of this binding may further clarify the role of m5C RNA modification in RAD52 recruitment to DNA damage sites.

It is also possible that intermediate factors are involved in RAD52 recruitment to DNA:RNA hybrids. It was reported that RAD52 recruitment requires Cockayne syndrome B protein (CSB), a key protein of transcription-coupled nucleotide-excision repair (Wei et al., 2015; Teng et al., 2018). These authors suggest that CSB recognizes DNA:RNA hybrids and then recruits RAD52 and RAD51C to DNA damage sites. However, the universality of this mechanism requires further investigation; reactive oxygen species used in this study as a source of DNA damage are known to generate multiple types of DNA damage including those that are specifically repaired by nucleotide excision repair (NER), which may not be common for other types of DNA damaging agents. Indeed, a CSB-independent mechanism of RAD52 recruitment has been reported (Tan et al., 2020). RAD52 is known to physically interact with other proteins involved in DNA repair, including RPA that stimulates the inverse RNA strand exchange activity of RAD52 (Mazina et al., 2017). RPA is a ubiquitous ssDNA binding protein, that was also found to bind ssRNA and to promote R-loop formation *in vitro* (Mazina et al., 2020). *In vivo*, RPA association with R-loops is well documented (Wei et al., 2015; Nguyen et al., 2017). It is possible that RPA is involved in RAD52 recruitment to DNA:RNA hybrids. Overall, the mechanism of RAD52 recruitment to transcriptionally active sites remains to be fully understood.

Upon its recruitment, RAD52 plays a pivotal role in the initiation of RNA-dependent DNA repair. RAD52 knockout in immortalized RPE-hTERT cells significantly reduced RPA and RAD51 foci formation after ionizing radiation and the rate of sister chromatid exchanges (Yasuhara et al., 2018). Importantly, recruitment of RAD51 to the sites of DNA damage was dependent on RAD52 specifically in transcriptionally active loci. Recent data indicate that RAD52 may also contribute to recruitment of POLD3, a subunit of DNA polymerase δ that is critical for BIR (Tan et al., 2020). Knockout of RAD52 in U2OS cells led to activation of NHEJ and increased chromosome aberrations indicating an important role of RAD52-mediated transcription-dependent DNA repair in the maintenance of genome stability.

Furthermore, RAD52 may play an important role in the resolution of DNA:RNA hybrids (or R-loops) by recruiting XPG nuclease, a member of the NER pathway (Yasuhara et al., 2018). These data together with the data by Lan’s group on interaction between RAD52 and CSB (Wei et al., 2015; Teng et al., 2018) indicate an intriguing crosstalk between the NER and HR pathways during DNA repair at active transcription sites. Moreover, in both of these studies, RAD52 plays a central role in linking HR and NER pathways during transcription-dependent DNA repair.

The relationship between the function of RAD52 in TA-HRR/TC-HR and its inverse RNA strand exchange activity raises an interesting question. Yasuhara et al., reported that the formation of DNA:RNA hybrids was not affected in a RAD52 knockout, arguing against the role of inverse RNA strand exchange activity of RAD52 in formation of these hybrids (Yasuhara et al., 2018). However, this study tracked formation of DNA:RNA hybrids during the initial 2 min response following DSB induction, whereas DSB repair *via* RAD52-mediated inverse RNA strand exchange likely requires an extended period of time comparable with a few hours as required for DSB repair *via* canonical HR. Therefore, it seems that RAD52 may play different roles at different stages of transcription-dependent DNA repair. In a rapid response, it may act by recruiting other DNA repair factors to the site of DNA damage at transcriptionally active sites, which parallels the mediator function of RAD52 in yeast where it promotes loading of RAD51 on RPA-covered ssDNA at the site of DNA damage (Sung 1997). While at later stages of DSB repair, RAD52 may promote formation of DNA:RNA hybrids in which RNA can be used as a template for DSB repair. The studies are currently under way to better understand the mechanisms of RNA-dependent DNA repair and the specific role(s) that RAD52 plays in this process.

### RAD52’s Role in Cancer Development

Cancer cells exhibit a high degree of DNA damage and genomic instability. It is known that BRCA1 and BRCA2 play important roles in HR-dependent repair of DSBs. However, BRCA-deficient tumors show increased dependence on alternative pathways such as SSA and a-EJ to overcome their “BRCAness” phenotype, characterized by reduced DSB repair, impaired replication fork protection, and hypersensitivity to DNA damaging agents (Stok et al., 2021). Through its strand annealing and DNA pairing activities, RAD52 is central to the SSA and BIR pathways (Gottifredi and Wiesmuller 2020). These alternative pathways are highly mutagenic and provide a conducive environment for chromosomal translocations to occur through non-specific, error-prone joining of two heterologous chromosomes (Malkova and Ira 2013; Blasiak 2021). For example, the hyper-resection of DSB ends in the absence of DNA damage sensor proteins such as 53BP1, DNA-PKcs, and EXOSC10 in S/G_2_ phase promotes mutagenic SSA activity (Domingo-Prim et al., 2019; Mladenov et al., 2019; Toma et al., 2019; Morales et al., 2021).

The hyper-mutagenic activity of BIR is primarily attributed to the significantly increased frequency of frameshift mutations, which may occur at a rate 2,800-fold higher than that of spontaneous mutations. These mutations are likely generated by the intermittent dissociation of Pol δ-synthesized DNA from its template during bubble migration (Sakofsky and Malkova 2017). This increases the propensity to incorporate mismatched nucleotides into the newly synthesized DNA which is normally repaired by mismatch repair (MMR) (Deem et al., 2011). However, the efficiency of MMR during BIR is significantly lower than that during S-phase replication. Another BIR-like mechanism, namely, alternative lengthening of telomeres (ALT) is implicated as a RAD52-dependent process involved in the development of human cancers (Sakofsky and Malkova 2017). One of the hallmarks of rapidly dividing cancer cells is their ability to efficiently maintain telomere length. While most cancer cells utilize telomerase to perform this activity, ∼15% of human cancers employ ALT. ALT-associated PML bodies contain telomeres, telomere-binding proteins, and the scaffold protein PML (Grobelny et al., 2000). RAD52 is required to promote ALT, and *in vitro* RAD52 can promote D-loop formation with telomeric ssDNA (Zhang et al., 2019). However, a RAD52-independent ALT pathway that relies on the endonuclease cofactor SLX4 has also been identified (Verma et al., 2019). Cells lacking both RAD52 and SLX4 are synthetically lethal due to the accumulation of genomic abnormalities, and thus are potential therapeutic targets in cancers that are telomerase deficient.

Several studies demonstrated that RAD52 is important for enhanced viability of cancer cells. The correlation between RAD52 overexpression and accelerated hepatocarcinogenesis in TGF-α/c-myc transgenic mice was the first significant evidence that highlighted the importance of RAD52 in tumor development (Hironaka et al., 2003). Deletion of RAD52 in an ATM-deficient background was shown to decrease T-cell lymphoma incidence and increase the life span of double-mutant mice (Treuner et al., 2004). ATM kinase activates cell cycle arrest, DNA repair, or apoptosis to restore proliferation of normal cells and maintain genomic stability, or eliminate heavily damaged cells. The loss of ATM kinase causes ataxia-telangiectasia, a syndrome associated with increased chromosomal abnormalities and high predisposition to breast cancer, brain cancer, lymphoma, and leukemia (Treuner et al., 2004; Estiar and Mehdipour 2018). Lieberman et al. showed that RAD52 deletion in Squamous Cell Lung Carcinoma increased the death of cells undergoing carcinogen-induced transformation *in vivo*. They also observed an increased antitumor activity in RAD52^-/-^ cells through an enhanced capacity of cytotoxic T lymphocytes and natural killer cells to directly kill tumor cells (Lieberman et al., 2017; Nogueira et al., 2019).

Several studies reported an association between high RAD52 expression level in tumor samples with poor patient prognosis and disease prognosis (Jewell et al., 2010; Lieberman and You 2017; Ho et al., 2020). In a study of cancer cells containing an inactivated RECQL4 gene and upregulated RAD52, inhibition of RAD52 sensitized the cancer cells to ionizing radiation (Kohzaki et al., 2020). Chronic expression of the CDK1 inhibitor p21 in pre-cancerous p53-deficient cells enables a subpopulation to develop with increased proliferation through deregulation of origin licensing during DNA replication (Galanos et al., 2016). It was shown that in these hyperproliferative cells, the p21-induced replication stress caused increased RAD52 expression and reliance on RAD52-dependent DNA repair pathways (Galanos et al., 2018). Further studies are needed to understand the contexts under which RAD52 expression can serve as a factor in determining the proper treatment to increase the success of patient outcomes.

### Synthetic Lethality and RAD52 as a Therapeutic Target

In normal cells, genome stability is maintained by a network of DDR pathways. Inactivation of DDR pathways due to intrinsic genome instability coerces tumor cells to rely on the remaining alternative DNA repair/signaling pathways. Not surprisingly, the pro-oncogenic role of RAD52 is especially pronounced in cancer cells that are deficient in DDR pathways, like ATM-deficient cancers (Treuner et al., 2004). But the most remarkable pro-cancer RAD52 phenotype is seen in cancer cells deficient in any of the following DNA repair proteins: BRCA1, BRCA2, PALB2, XAB2 or RAD51 paralogs: RAD51B, RAD51C, RAD51D, XRCC2 and XRCC3 (Feng et al., 2011; Chun et al., 2013; Lok et al., 2013; Sharma et al., 2021). Powell’s group showed that cells in which one of these proteins were mutated or depleted became dependent on RAD52 for viability; thus, mutations in RAD52 are synthetically lethal with mutations/depletion in these proteins. The term synthetic lethality refers to scenarios in which the simultaneous disruption of two biological pathways results in cell death, but disruption of either one in isolation does not (Dobzhansky 1946). It was proposed that RAD52 operates in a DSB repair sub-pathway that is distinct from the major BRCA-dependent HR pathway (Jalan et al., 2019). Recent data indicate that RAD52 “catalytic” activities encoded by the N-terminal domain which include DNA pairing (D-loop formation), ssDNA and RNA annealing, inverse RNA and DNA strand exchange; are responsible for the viability of BRCA-deficient cells (Hanamshet and Mazin 2020). Which of these specific activities of RAD52 that are critical for the viability remains to be identified. The role of the C-terminal domain and its potential mediator function (similar to yeast Rad52) with RAD51 and RPA, remains to be investigated as well.

Hromas with co-workers showed RAD52/BRCA synthetic lethality depends on EEPD1, a structure-specific endonuclease that cleaves stalled replication forks (Hromas et al., 2017). Depletion of EEPD1 suppressed the synthetic lethality of RAD52-depleted BRCA1^-^ cells, as DNA breaks are shunted toward (or processed by) the a-EJ pathway. Thus, the synthetic lethal relationship between BRCA and RAD52 is dependent on the generation of dead-end DNA intermediates that no remaining DNA repair pathway can handle in BRCA- and RAD52-deficient cells.

The synthetically lethal relationship between RAD52 and BRCA-related genes has important practical implications because mutations in BRCA1/2 and several related genes are responsible for nearly half of familial breast and ovarian cancers. Adamson et al. recently showed in populational studies that a RAD52 S346X polymorphic variant significantly reduces breast cancer risk among germline BRCA2 mutation carriers. This variant encodes a truncated RAD52 lacking the last 8 amino acids composing a nuclear localization signal. Cytoplasmic retention renders this RAD52 variant nonfunctional leading apparently to attrition of BRCA2-deficient breast cancer cells (Adamson et al., 2020; Biswas and Sharan 2020).

Targeting DNA repair proteins in synthetically lethal relationships has emerged as a prime strategy of novel cancer therapeutics (Huang et al., 2020; Myers et al., 2020). Thus, inhibitors of the DNA repair protein PARP represent the newest generation of cancer therapeutics (Lord and Ashworth 2017; D'Andrea 2018). However, the majority of cancer patients treated with PARP inhibitors (PARPi) eventually develop resistance to these agents, which stresses the need for new therapeutics (Lord and Ashworth 2013). Because in humans, RAD52 mutations cause no discernible HR phenotype, the synthetically lethal BRCA/RAD52 relationship makes RAD52 an attractive therapeutic target.

**FIGURE 4:**
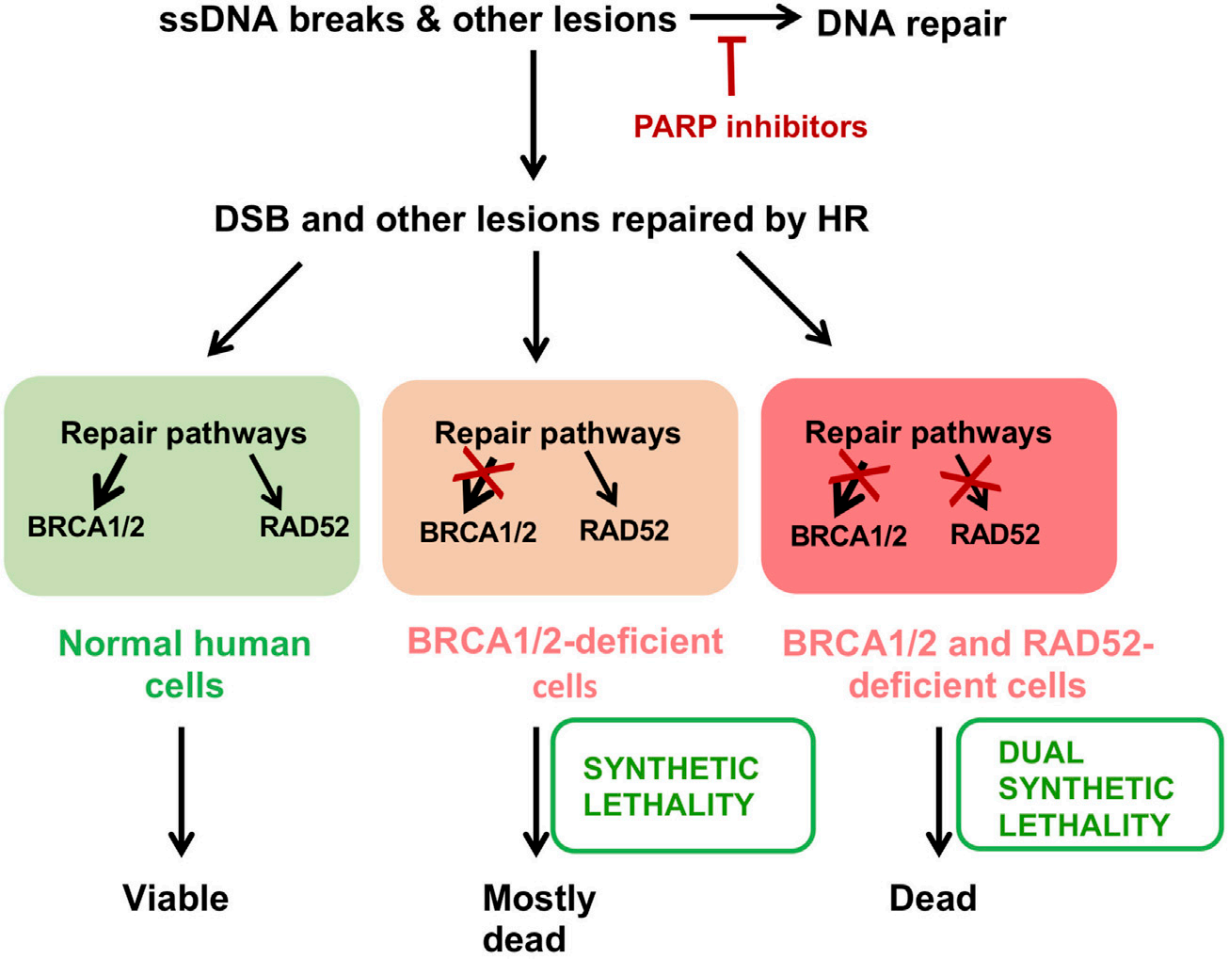
Targeting cancer cells *via* synthetic lethality. PARP inhibitors trap PARP on DNA lesions and suppress repair of ssDNA breaks. This leads to generation of DSBs and other lesions that can only be repaired by HR. Normal cells are capable of repairing these lesions. Dysfunction of BRCA1/2 and related genes cause synthetic lethality with PARP inhibitors so that most of these cells die. Selective pressure forces the cancer cells to become more dependent on alternative RAD52-dependent DNA repair pathways. A combinational treatment of PARP and RAD52 inhibitors enhances the efficacy of each individual treatment *via* dual synthetic lethality and may cause a delay in the development of cancer drug resistance.

The synthetically lethal relationship between RAD52 and BRCA was first exploited using an oligopeptide aptamer to inhibit RAD52 in BRCA-downregulated acute myeloid leukemia cells. As expected, these cells arrested in G_2_ and showed increased apoptosis (Cramer-Morales et al., 2013; Xu et al., 2020). Later, our and several other groups developed small molecule RAD52 inhibitors to specifically suppress the growth of BRCA-deficient cancer cells (Chandramouly et al., 2015; Hengel et al., 2016; Huang et al., 2016; Sullivan et al., 2016; Hengel et al., 2017; Sullivan-Reed et al., 2018). One of these compounds, D-I03, showed anti-proliferative activity against BRCA1-deficient breast cancer cells both *in vitro* and *in vivo* (Sullivan-Reed et al., 2018). However, the highest anti-proliferating activity of D-I03 was observed in combination with the PARP inhibitor Talazoparib. This is consistent with the different mechanisms of action of PARP and RAD52 inhibitors. While PARP inhibitors increase the DNA damage load for the HR pathway and inhibit alternative a-EJ pathway, RAD52 inhibitors block the escape route for BRCA-deficient cancer cells through RAD52-dependent mechanism(s) of DNA repair (Figure 4). Combination treatment may also help to attenuate formation of drug resistance in cancer, the main nemesis of anti-cancer therapies. More work is needed for development of truly drug-like RAD52 inhibitors that can be used in clinic.

## Conclusions and Future Perspectives

In yeast, Rad52 is a key protein of HR. The biochemical studies show that it may play a mediator function by assisting Rad51 recombinase loading on ssDNA occupied by RPA. But these studies may not tell the whole story, as genetic data indicate a stronger Rad52 phenotype in DSB repair and HR than that of Rad51 recombinase. In contrast to yeast, mammalian RAD52 knockouts show a mild phenotype in DNA repair and recombination in otherwise normal cells. However, RAD52 function became essential for viability of BRCA-deficient cancer cells. RAD52 is a multifunctional protein with several important activities including DNA pairing (D-loop formation) and ssDNA annealing. Recent studies uncovered an important role of RAD52 in RNA-dependent DNA repair and in R-loop resolution. RAD52 can promote DNA:RNA annealing and inverse strand exchange between RNA and homologous dsDNA at the proximity DSBs. Determining which of these activities play a critical role for viability of BRCA-deficient cancer cells remains a subject of investigation. Better understanding of RAD52 function will clarify the mechanisms of DNA repair in eukaryotes, and in humans particularly. Importantly, the synthetically lethal RAD52/BRCA relationship provides an opportunity to develop new anti-cancer drugs targeting BRCA-deficient cancers. Use of these inhibitors in combination with PARP inhibitors or other targeted therapies is a promising approach to increase the efficacy of the treatment and attenuate formation of drug resistance in cancer.
